# Application of bone metabolic parameters in the diagnosis of growing pains

**DOI:** 10.1002/jcla.24184

**Published:** 2021-12-24

**Authors:** Huamei Li, Bing Wang, Lin He, Ran Tao, Shiqiang Shang

**Affiliations:** ^1^ Department of Clinical Laboratory The Children’s Hospital Zhejiang University School of Medicine National Clinical Research Center For Child Health Hangzhou China; ^2^ Department of Endocrinology The Children’s Hospital Zhejiang University School of Medicine National Clinical Research Center For Child Health Hangzhou China; ^3^ Zhejiang University School of Medicine Hangzhou China

**Keywords:** bone metabolism, diagnosis, growing pains, parameters

## Abstract

**Objective:**

The present study aimed to assess the diagnostic significance of serum bone metabolic parameters in children with growing pains (GPs).

**Methods:**

All patients diagnosed with GP and healthy controls matched with age and gender were recruited at the outpatient clinic of Children's Hospital at Zhejiang University School of Medicine from August 2016 to August 2021. In all subjects, serum levels of calcium (Ca), phosphorus (P), procollagen type‐I N‐terminal (PINP), parathormone (PTH), 25‐hydroxyvitamin D (25‐(OH)D), osteocalcin (OC), N‐terminal cross‐linked telopeptides of type‐I collagen (CTX), and tartrate‐resistant acid phosphatase type 5b (TRACP5b) were investigated. The univariate analysis, multivariate logistic regression analysis, and receiver operating characteristic (ROC) curve were used to identify the bone metabolic parameters factors for diagnosing GP.

**Results:**

We enrolled 386 children with GP and 399 healthy controls in present study. The mean age of GP group was 5.319 years, and, primarily, the subjects were preschool‐age children. The gender ratio (male‐to‐female) was 1.27 in GP group. After adjusting for age and gender, we identified that the serum levels of Ca (*p* < 0.001, OR: 25.039), P (*p* = 0.018, OR: 2.681), PINP (*p* < 0.001, OR: 1.002), and PTH (*p* = 0.036, OR: 0.988) were independent diagnostic factors associated with GP. Area under curve (AUC) of the ROC curves was in the order: PINP (0.612) > Ca (0.599) > P (0.583) > PTH (0.541). A combination of independent diagnostic factors and multivariable logistic regression analysis provided a refined logistic regression model to improve the diagnostic potential, of which the AUC had reached 0.655.

**Conclusions:**

Serum levels of Ca, P, PINP, and PTH could be independent diagnostic factors associated with GP. The logistic model was significantly superior to bone metabolic parameters for diagnosing GP.

## Graphical abstract

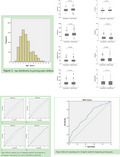

## 1 INTRODUCTION

Growing pains (GPs) were first described in 1823 by French physician Marcel Duchmap as the most common recurrent leg pain problem in children, which occur in about 3% to 47% of children with no organic lesions.^1^, ^2^ There is no evidence that GP is associated with particularly rapid growth.^3^ GP mostly affects children aged 3–12 years, and the highest incidence is found in the 4‐ to 6‐year age bracket.^3^ GP is almost always bilateral, usually nonarticular, and often located in the shins, calves, thighs, or popliteal fossa.^4^ The pain usually occurs at night, with intensity varying from mild to very severe, and resolves by morning. The severity and duration of pain varies from child to child.^5^ In severe cases, the pain can occur daily. The duration of pain ranges from minutes to hours. Some children experience regular pain, whereas others have days or months of pain‐free periods between episodes, which vary from child to child.^6^ Previous studies found association between GP and abdominal pains, nonspecific headaches, and behavioral disorders.^7^ Meanwhile, GP is not related to serious organic lesions and usually resolves by late childhood. The physical examination showed no objective signs of inflammation.^8^, ^9^ However, frequent pain may have tremendous impacts on the children and their family's daily routines including activity, sleep, frequent use of pain relief medications, and causing anxiety.^10^, ^11^ The etiology of GP has still not been fully elucidated, despite its discovery for over 200 years ago. Many different theories have been postulated, but none have been confirmed. Unknown etiology contributes to diagnostic difficulty that the diagnosis of GP requires the exclusion of other diseases with similar clinical manifestations.^12^ Thereby, GP has been more of a diagnosis of exclusion than an explicit entity with ambiguity in diagnostic criteria, which has become a hot research topic.

According to some studies, the bone mineral density of some children with GP was significantly less than that of healthy children, and hypovitaminosis D may play a role in the pathogenesis of GP.^4^, ^13^ Therefore, we speculate that altered bone metabolism may occur in children with GP. Traditionally, densitometry techniques were recognized as a gold standard for the assessment of bone status. However, these measures only reflect the static state of bone tissue.^14^ The detection of bone metabolism markers can reflect the dynamic state of bone metabolism, and they show a significantly higher sensitivity at the early stage of bone metabolism changes.^15^ Metabolites released by osteoblasts during bone formation and bone matrix fragments and secretions produced by osteoclasts during bone resorption enter the bloodstream and can be detected. They are divided into three types: markers for bone formation, markers for bone resorption, and calcium (Ca) and phosphorus (P) metabolism indicators. For bone formation, markers reflecting osteoblast activity are by‐products of osteoblastic enzymes, matrix proteins, or collagen synthesis, including procollagen type‐I N‐terminal (PINP) released during the procession of type‐I procollagen into collagen and osteocalcin (OC) synthesized by mature osteoblasts.^16^, ^17^, ^18^ For bone resorption, markers reflecting osteoclast activity are degradation products of type‐I collagen, such as C‐terminal cross‐linked telopeptides of type‐I collagen (CTX) and tartrate‐resistant acid phosphatase type 5b (TRACP5b) which is an enzyme secreted by osteoclasts.^13^ Calcium and phosphorus metabolism indicators mainly include 25‐hydroxyvitamin D (25(OH)D), parathormone (PTH), calcium (Ca), and phosphorus (P). The homeostasis of calcium and phosphate is mainly regulated by 25(OH)D and PTH, promoting bone mineralization.^19^ Advances in the correlation between the above bone metabolic parameters and GP may provide potential alternative diagnostic targets in diagnostic studies of GP.

The study aimed to investigate differential bone metabolism parameters between GP and healthy children, through measuring the serum levels of markers of bone reformation (PINP and OC), markers of bone absorption (CTX and TRACP5b), and calcium and phosphorus metabolism indicators (25(OH)D, PTH, Ca, and P). Analyzing diagnostic value of bone metabolic parameters will complement the currently utilized exclusion‐based diagnostic strategies of GP.

## 2 MATERIALS AND METHODS

### 2.1 Patients

This study was conducted at the Children's Hospital of Zhejiang University School of Medicine, from August 2016 to August 2021. The children with GP were diagnosed and confirmed according to the criteria defined by Petersen. The inclusion criterion of GP was children affected by intermittent (nonarticular) pains in both legs, generally occurring late in the day or at night, with no signs of inflammation. Exclusion criteria were listed as following: a history of bone disease; persistent pain; physical signs (swelling, local tenderness, redness, trauma, movement limitation, and limping) and laboratory examinations of articular diseases (blood tests and imaging); treatment with drugs that could affect bone metabolism (i.e., glucocorticoid, immunosuppressant, anticonvulsants, and antirejection medications); illnesses such as malnutrition, rickets, rheumatologic disorders, celiac disease, or systemic illness; and taking vitamin or mineral supplements.^20^, ^21^ Meanwhile, unrelated healthy controls matched for age and gender were recruited from the outpatients of our hospital who were diagnosed with no symptoms of GP or any diseases that might affect bone metabolism. Written consents were obtained from subjects’ guardian. The study was approved by the Ethics Committee of the Children's Hospital of Zhejiang University School of Medicine.

### 2.2 Laboratory tests

Fasting venous blood were drawn from all subjects in the morning. We measured serum concentrations of CTX and TRACP5b as bone resorption indexes and serum concentrations of PINP and OC (we tested “the amino‐terminal and the midsection OC, N‐MID‐OC”) as bone formation indexes. Serum concentrations of 25(OH)D and PTH, Ca, and P were also measured. Serum concentrations of 25(OH)D were detected by enzyme‐linked immunosorbent assay (ELISA) (ids isys, England) and TRACP5b by ELISA (PHICON, China). Serum concentrations of CTX, PINP, OC, and PTH were visualized using chemiluminescence immunoassay analyzer (CLIA) (ids isys, England). Limits of detection were 0.7 U/L for TRACP5b, 6.8 nmol/L for 25(OH)D, 0.023 ng/ml for CTX, 1.0 ng/ml for PINP, 0.27 ng/ml for OC, and 2.5 pg/ml for PTH. Serums Ca and P were measured by a standardized colorimetric test (Beckman AU5800 USA). All assays were performed according to the manufacturer's instructions.

### 2.3 Statistical analysis

Data analysis was performed using SPSS software (SPSS 19.0). Plots of the study were generated using GraphPad Prism 6.0 software. Quantitative variables were reported as mean and 95% confidence interval (95% CI). Independent samples *T* test or nonparametric test was used to compare means between children with GP and controls. Pearson's Chi‐square test was used to analyze the categorical variables. Multivariate logistic regression analysis was used to identify independent predictors and construct the refined logistic regression model for the diagnostic of GP. The cut‐off values of bone metabolic parameters were defined by Youden indices shown in receiver operating characteristic (ROC) curve analysis. The diagnostic performance of bone metabolic parameters was measured using the area under ROC curve (AUC). The criterion for statistical significance was *p* < 0.05.

## 3 RESULTS

### 3.1 Demographic data

**FIGURE 1:**
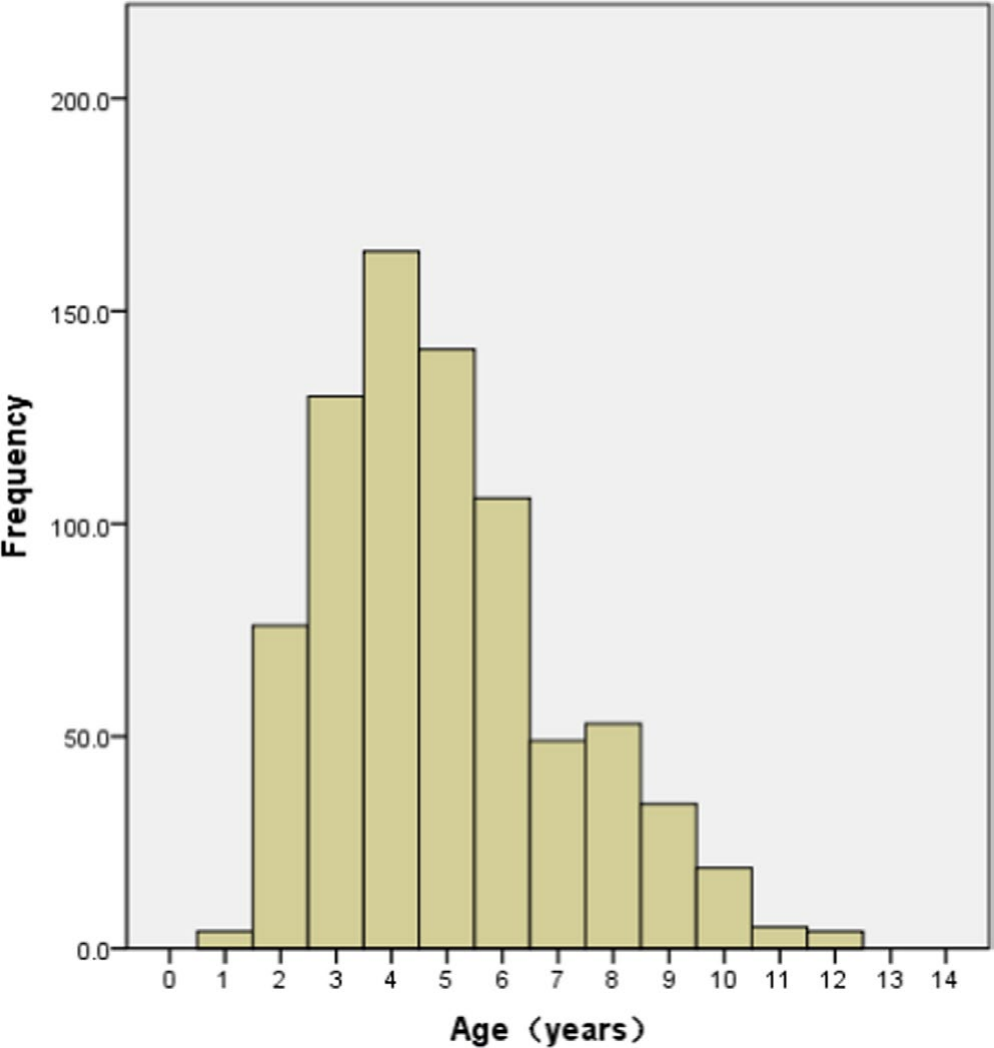
Age distribution in growing pains children

From August 2016 to August 2021, a total of 386 children with GP were collected, which included 216 boys and 170 girls and the male‐to‐female ratio was 1.27. The mean age of the study population was 5.319 (5.118, 5.520) years (range 1–12 years). According to age,^22^ the subjects were divided into four subgroups: 0–2 years old (infancy and childhood), 3–6 years old (preschool age), 7–12 years old (school age), and more than 12 years old (puberty). Children with GP were primarily preschool‐age children (53.6%), followed by infancy and childhood children (27.5%), and no patient in puberty. Age distribution in GP children is presented in Figure 1. Furthermore, we recruited 399 unrelated healthy controls in which included 232 boys and 167 girls and the male‐to‐female ratio was 1.39. The mean age of controls was 5.427 (5.222, 5.632) years (range 1–12 years). There was no significant difference in age (*Z* = −0.817, *p* = 0.414) and gender (χ^2^ = 0.383, *p* = 0.536) between the GP and the healthy controls. There was no significant difference in gender between the GP and the healthy controls in three age subgroups (all *p* > 0.05).

### 3.2 Description of bone metabolic parameters

**FIGURE 2:**
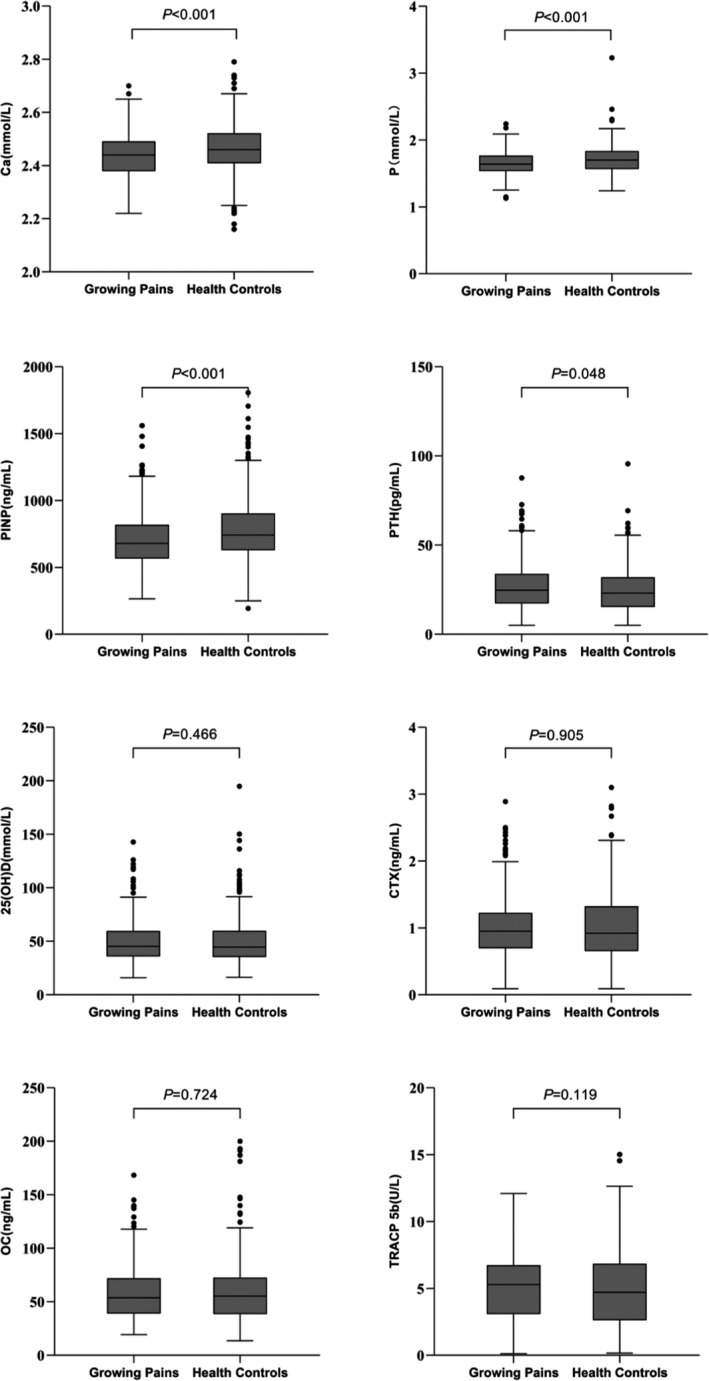
Comparisons of parameters related to bone metabolism in different groups

Comparisons of parameters related to bone metabolism in different groups are presented in Figure 2. In the univariate analysis, serum levels of Ca (*Z* = −4.710), P (*Z* = −4.060), or PINP (*Z* = −5.310) in GP group were significantly lower than in healthy controls (all *p* < 0.05). Rather, serum level of PTH was higher in GP group than in healthy controls (*Z* = −1.981, *p* < 0.05), and there was no significant difference in serum OC, CTX, TRACP5b, and 25‐(OH)D levels between GP group and healthy controls (all *p* > 0.05).

### 3.3 Diagnostic value of bone metabolic parameters

For diagnosis of GP, a multivariate logistic regression analysis was performed to assess significant bone metabolic parameters variables with *p* < 0.1 which were derived from the univariate analysis. After adjusting for age and gender factors, the present study identified that serum Ca level (*p* < 0.001, Odds ratio (OR): 25.039), P level (*p* = 0.018, OR: 2.681), PINP level (*p* < 0.001, OR: 1.002), and PTH level (*p* = 0.036, OR: 0.988) were independent predictors for the diagnostic of GP. Further details of relevant data are stated in Table 1.

**TABLE 1:** Multivariate logistic regression analysis for diagnosis of growing pains (GP)

	GP group (*N* = 386)	Health controls (*N* = 399)	Beta value	OR	95% CI	*p* value
Male gender (%)	216 (56.0%)	232 (58.15%)	−0.177	—	—	0.250
Age (years)	5.319 (5.118, 5.521)	5.427 (5.222, 5.632)	0.062	—	—	0.100
CA	2.435 (2.426, 2.444)	2.469 (2.459, 2.477)	3.468	25.039	(4.494, 139.497)	<0.001
P	1.648 (1.630, 1.666)	1.707 (1.688, 1.727)	5.644	2.681	(1.170, 6.144)	0.018
PINP	700.179 (680.417, 719.942)	782.977 (759.953, 806.000)	21.639	1.002	(1.001, 1.002)	<0.001
PTH	26.359 (25.072, 27.645)	24.577 (23.367, 25.787)	4.418	0.988	(0.976, 0.999)	0.036

Abbreviations: CA, calcium; CI, confidence interval; P, phosphorus; PINP, procollagen type‐I N‐terminal; PTH, parathormone; OR, odds ratio.

### 3.4 The role of bone metabolic parameters in diagnosing GP

**FIGURE 3:**
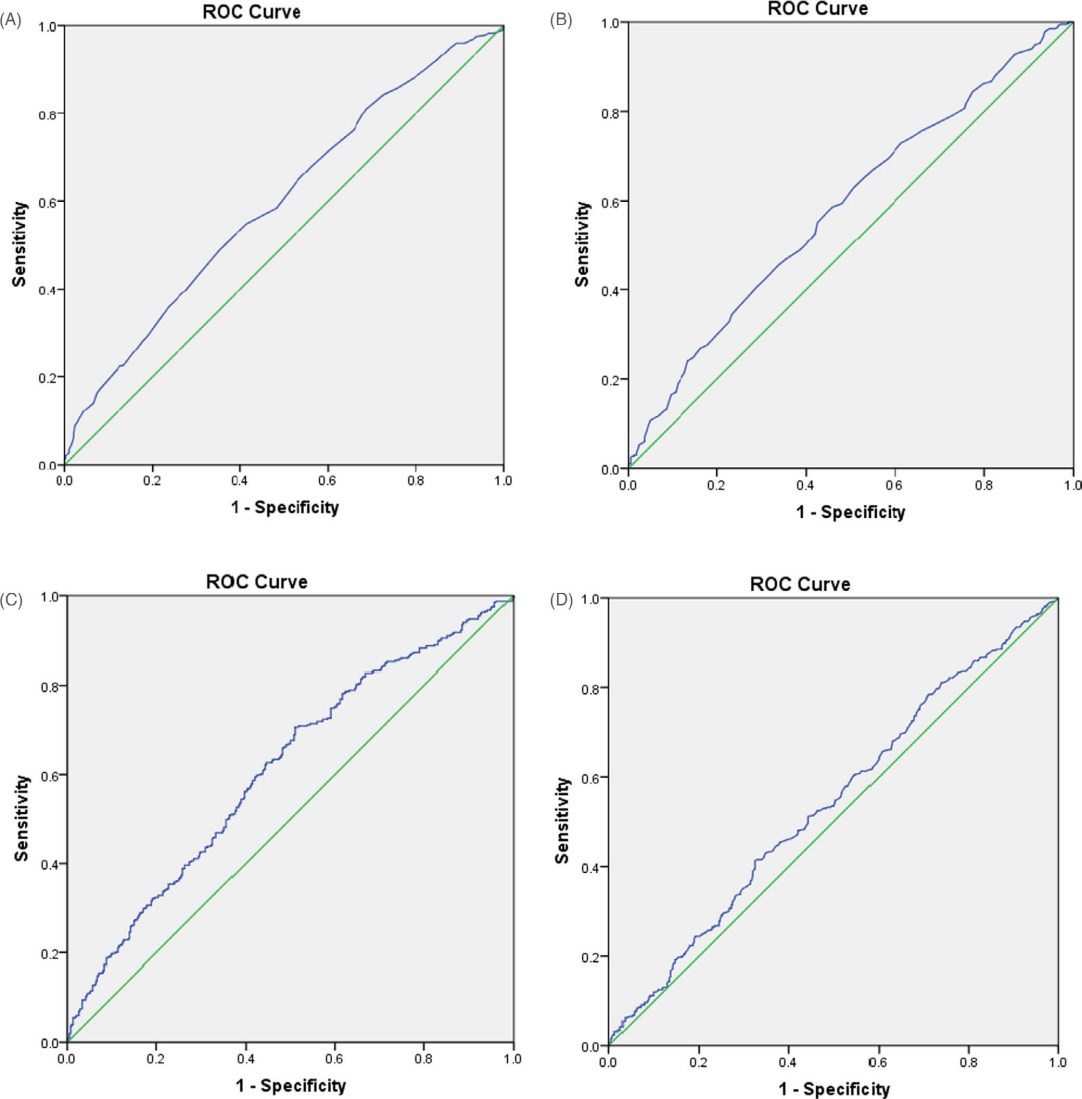
Receiver operating curve (ROC) of four independent predictors for the diagnostic of growing pains. The predictors were Ca (A), P (B), PINP (C), and PTH (D)

Diagnostic performance of bone metabolic parameters was assessed by applying the ROC curve. As is shown in Figure 3, area under ROC curve was the highest for PINP level, followed by Ca level, P level, and PTH level. With the utilization of ROC curve and Youden index, the cut‐off values of serum PINP, Ca, P, and PTH levels for diagnosing GP were determined. Relevant sensitivity and specificity at the maximum of the Youden index were calculated and shown in Table 2.

**TABLE 2:** Predictive accuracies of ROC characteristics in diagnosing children with growing pains

	Ca	P	PINP	PTH
Area under ROC curve	0.599	0.583	0.612	0.541
Cut‐off value	2.465	1.665	664.435	27.8
Sensitivity (%)	48.9	57.4	70.8	41.3
Specificity (%)	64.9	55.3	49.1	67.5
Youden index	0.138	0.127	0.199	0.088

Abbreviations: CA, calcium; P, phosphorus; PINP, procollagen type‐I N‐terminal; PTH, parathormone; ROC, receiver operating characteristic.

**FIGURE 4:**
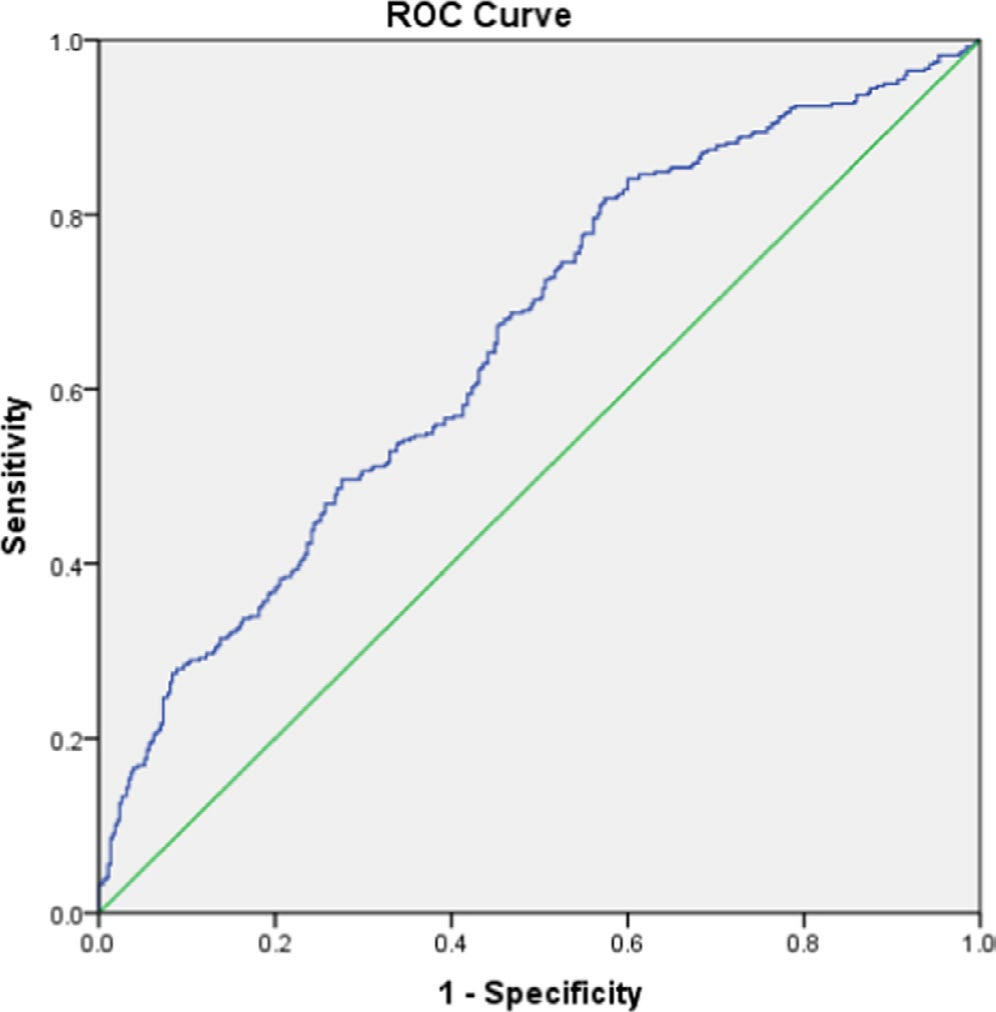
Receiver operating curve (ROC) of logistic model for diagnosing growing pains

Furthermore, a refined logistic regression model was built as a prediction model for the diagnostic of GP, which was based on the above results of multivariable logistic regression analysis. The best diagnostic logistic regression model is Logit(*P*) = −10.4510 + 3.220 × Ca +0.9868 × P + 0.002 × PINP −0.012 × PTH. The predicated probability of diagnosing GP for each subject was *P* = e^Logit(P)^ / 1 + e^Logit(P)^. The area under ROC curve of predictive model was 0.655, which was higher than each single bone metabolic parameter for diagnosing GP (Figure 4). Optimum cut‐off value was 0.427, according to the Youden index. Relevant sensitivity and specificity were, respectively, 81.9% and 42.6% at the maximum of the Youden index (0.245).

## 4 DISCUSSION

Many theories have been put forward as to the etiology of GP. The main theories include low pain threshold, less bone strength density, blood perfusion changes, anatomical or mechanical, family environment, a manifestation of an organic disease like metabolic muscle disease or restless leg syndrome, the content of some trace elements, hypovitaminosis D, and perinatal risk factors,^10^, ^23^, ^24^, ^25^, ^26^ but none has been confirmed. Because the cause is obscure, the diagnosis of GP itself is based on exclusion rather than definite evidence.

Bone undergoes a constant remodeling process via old bone resorption by osteoclasts and new bone formation by osteoblasts throughout life.^27^ The bone metabolism rate can be evaluated noninvasively by measuring specific markers of bone resorption and bone formation in serum. According to our research in literature, there is no other study about bone metabolism and GP with similar magnitude. The study showed that children with GP have lower levels of PINP than healthy controls. The result which indicated the function of osteoblasts may be decreased in patients of GP. This mean GP maybe is not a normal physiological phenomenon but related to decreased bone metabolism. However, there were no significantly lower values for other bone formation indicators in children with GP. These results suggest that PINP may be more sensitive than other indicators to reflect the role of bone formation in GP. Ca and P are generally considered the reflection of the bone homeostasis. Ca supplementation may increase bone strength density.^13^ Simon found that Ca supplementation may ease GP.^28^ Qamar et al. also found that a minority of children with GP have hypocalcemia (6%) and hypophosphatemia (3%) in their study,^4^ but there was also no control group used for hypothesis tested. In this study, we found that serum concentrations of Ca and P were significantly decreased in GP than healthy controls that supported Simon's view. In addition, the present study indicated that PTH is an independent predictor for GP, and it is also an important regulator of Ca and P levels. This may suggest that the mechanism that PTH involved in the disease GP is not merely achieved by affecting Ca or P level, relevant mechanism needs to be investigated in detail.

With the change of growth rate, the serum concentration of bone markers varied with age and sex.^29^ Bone formation parameters increase in infancy, and bone formation and bone resorption parameters increase in both sexes during adolescence.^30^ In view of this, age and gender were considered for inclusion in the multivariate regression modeling analysis. After having adjusted for age and gender, bone formation‐related marker PINP and calcium and phosphorus metabolism indicators of PTH, Ca, and P remained independent predictors for GP diagnosis.

In this study, bone resorption markers of CTX and TRACP5b were not associated with GP. This suggests that bone resorption is not involved in the pathogenesis and course of GP, and relevant research was rather scarce. In our case–control study, no association was found between vitamin D (VD) levels and GP. However, the theory that VD deficiency is related to GP is often mentioned. In a study including 120 children with GP, 86.6% cases were found to be deficient in VD. After 3 months of oral vitamin supplementation, the level of 25(OH)D was significantly increased, and the pain score was significantly decreased.^25^ Morandi et al. also found that VD levels were significantly increased and pain intensity significantly decreased by VD supplementation in children with GP.^21^ However, one drawback of these studies was that there was no control group for hypothesis test or some studies have smaller sample sizes.^3^ In addition, because GP is a self‐limiting disease, the appropriateness of comparing pain scores over several months is questionable. Together, the results of this study showed no difference in association between VD levels and GP.

This study assessed the diagnostic value of PINP, Ca, P, and PTH in GP and found that the combination of the above indicators constructs a logistic regression model with higher diagnostic performance. To our knowledge, this is the first reported attempt to develop a diagnostic model of GP based on bone metabolic parameters. The results of this study are helpful to shift the diagnosis of GP from a diagnosis based on exclusion to an active and logical search for diagnostic indicators. The follow‐up clinical practice application with large sample size of this research model needs to be carried out, which will provide more valuable evidence for the standardization of GP diagnosis.

## 5 CONCLUSIONS

In conclusion, the present study suggests that bone metabolism parameters including serum levels of PINP, Ca, P, and PTH were independent diagnostic factors associated with GP. The logistic model was significantly superior to the single bone metabolism parameter for diagnosing GP.

## CONFLICT OF INTEREST

The authors declare that they have no conflict of interests.

## AUTHOR CONTRIBUTIONS

Huamei Li and Bing Wang designed the research and wrote the article. Huamei Li, Bing Wang, Lin He, and Ran Tao completed the experimental part of the study, collected the laboratory parameters and patients’ information, performed the statistical analysis, and drew the figures. Shiqiang Shang supervised the entire study and provided academic guidance throughout the study process.

## Data Availability

The data that support the findings of this study are available from the corresponding author upon reasonable request.

## References

[1] Bowyer SL , Hollister JR . Limb pain in childhood. Pediatr Clin North Am. 1984;31(5):1053‐1081. 6384901 10.1016/s0031-3955(16)34684-3

[2] Margaret Evans A , Doreen Scutter S . Prevalence of "growing pains" in young children. J Pediatr. 2004;145(2):255‐258. 15289780 10.1016/j.jpeds.2004.04.045

[3] Lehman PJ , Carl RL . Growing pains. Sports Health. 2017;9(2):132‐138. 28177851 10.1177/1941738117692533 PMC5349398

[4] Qamar S , Akbani S , Shamim S , Khan G . Vitamin D levels in children with growing pains. J Coll Physicians Surg Pak. 2011;21(5):284‐287. 21575536

[5] Uziel Y , Hashkes PJ . Growing pains in children. Pediatr Rheumatol Online J. 2007;5:5. 17550631 10.1186/1546-0096-5-5 PMC1869025

[6] Harel L . Growing pains: myth or reality. Pediatr Endocrinol Rev. 2010;8(2):76‐78. 21150836

[7] Halliwell P , Monsell F . Growing pains: a diagnosis of exclusion. Practitioner. 2001;245(1624):620‐623. 11464551

[8] Asadi‐pooya AA , Bordbar MR . Are laboratory tests necessary in making the diagnosis of limb pains typical for growing pains in children? Pediatr Int. 2007;49(6):833‐835. 18045281 10.1111/j.1442-200X.2007.02447.x

[9] Evans AM . Growing pains: contemporary knowledge and recommended practice. J Foot Ankle Res. 2008;1(1):4. 18822152 10.1186/1757-1146-1-4 PMC2553776

[10] Weiser P . Approach to the patient with noninflammatory musculoskeletal pain. Pediatr Clin North Am. 2012;59(2):471‐492. 22560580 10.1016/j.pcl.2012.03.012

[11] Lowe RM , Hashkes PJ . Growing pains: a noninflammatory pain syndrome of early childhood. Nat Clin Pract Rheumatol. 2008;4(10):542‐549. 18762787 10.1038/ncprheum0903

[12] Mohanta MP . Growing pains: practitioners’ dilemma. Indian Pediatr. 2014;51(5):379‐383. 24953579 10.1007/s13312-014-0421-0

[13] Friedland O , Hashkes PJ , Jaber L , et al. Decreased bone speed of sound in children with growing pains measured by quantitative ultrasound. J Rheumatol. 2005;32(7):1354‐1357. 15996077

[14] Jürimäe J . Interpretation and application of bone turnover markers in children and adolescents. Curr Opin Pediatr. 2010;22(4):494‐500. 20508524 10.1097/MOP.0b013e32833b0b9e

[15] Barera G , Beccio S , Proverbio MC , Mora S . Longitudinal changes in bone metabolism and bone mineral content in children with celiac disease during consumption of a gluten‐free diet. Am J Clin Nutr. 2004;79(1):148‐154. 14684411 10.1093/ajcn/79.1.148

[16] Eastell R , Hannon RA . Biomarkers of bone health and osteoporosis risk. Proc Nutr Soc. 2008;67(2):157‐162. 18412989 10.1017/S002966510800699X

[17] Szulc P , Kaufman JM , Delmas PD . Biochemical assessment of bone turnover and bone fragility in men. Osteoporos Int. 2007;18(11):1451‐1461. 17566813 10.1007/s00198-007-0407-z

[18] Risteli L , Risteli J . Biochemical markers of bone metabolism. Ann Med. 1993;25(4):385‐393. 8217105 10.3109/07853899309147301

[19] Khundmiri SJ , Murray RD , Lederer E . PTH and Vitamin D. Compr Physiol. 2016;6(2):561‐601. Published 2016 Mar 15. 27065162 10.1002/cphy.c140071 PMC11163478

[20] Peterson HA . Leg aches. Pediatr Clin North Am. 1977;24(4):731‐736. 927937 10.1016/s0031-3955(16)33494-0

[21] Morandi G , Maines E , Piona C , et al. Significant association among growing pains, vitamin D supplementation, and bone mineral status: results from a pilot cohort study. J Bone Miner Metab. 2015;33(2):201‐206. 24633492 10.1007/s00774-014-0579-5

[22] Jiang ZF , Shen KL , Shen Y . Zhu Futang Practice of Peditrics. 8th ed. People's Medical Publishing House.

[23] Braegger C , Campoy C , Colomb V , et al. Vitamin D in the healthy European paediatric population. J Pediatr Gastroenterol Nutr. 2013;56(6):692‐701. 23708639 10.1097/MPG.0b013e31828f3c05

[24] Lech T . Lead, copper, zinc, and magnesium content in hair of children and young people with some neurological diseases. Biol Trace Elem Res. 2002;85(2):111‐126. 11899019 10.1385/BTER:85:2:111

[25] Vehapoglu A , Turel O , Turkmen S , et al. Are growing pains related to vitamin D deficiency? Efficacy of vitamin D therapy for resolution of symptoms. Med Princ Pract. 2015;24(4):332‐338. 26022378 10.1159/000431035 PMC5588252

[26] Kaspiris A , Chronopoulos E , Vasiliadis E . Perinatal risk factors and genu valgum conducive to the onset of growing pains in early childhood. Children (Basel). 2016;3(4):34. 10.3390/children3040034 PMC5184809 27869739

[27] Manolagas SC , Jilka RL . Bone marrow, cytokines, and bone remodeling. Emerging insights into the pathophysiology of osteoporosis. N Engl J Med. 1995;332(5):305‐311. 7816067 10.1056/NEJM199502023320506

[28] Simon MW . Growing pains in children: solved and resolved. Clin Pediatr (Phila). 2015;54(7):706. 25527530 10.1177/0009922814563928

[29] Van Coeverden SC , Netelenbos JC , de Ridder CM , Roos JC , Popp‐Snijders C , Delemarre‐van de Waal HA . Bone metabolism markers and bone mass in healthy pubertal boys and girls. Clin Endocrinol (Oxf). 2002;57(1):107‐116. 12100078 10.1046/j.1365-2265.2002.01573.x

[30] Geserick M , Vogel M , Eckelt F , et al. Children and adolescents with obesity have reduced serum bone turnover markers and 25‐hydroxyvitamin D but increased parathyroid hormone concentrations ‐ Results derived from new pediatric reference ranges. Bone. 2020;132:115124. 31830529 10.1016/j.bone.2019.115124

